# Arthroscopic patellar release for treatment of chronic symptomatic patellar tendinopathy: long-term outcome and influential factors in an athletic population

**DOI:** 10.1186/s12891-017-1851-3

**Published:** 2017-11-22

**Authors:** Gernot Lang, Jan M. Pestka, Dirk Maier, Kaywan Izadpanah, Norbert Südkamp, Peter Ogon

**Affiliations:** 1 Department of Orthopedics and Trauma Surgery, University Medical Center Freiburg, Albert-Ludwigs-University of Freiburg, Faculty of Medicine, Hugstetter Strasse 55, 79106 Freiburg, Germany; 2 Center of Orthopedic Sports Medicine Freiburg, Freiburg, Germany

**Keywords:** Knee, Pain, Risk, Patellar, Tendinitis, jumper’s knee, MRI, Prognosis, Outcome, Return to sports

## Abstract

**Background:**

Arthroscopic patellar release (APR) is utilized for minimally invasive surgical treatment of patellar tendinopathy. Evidence regarding long-term success following the procedure is limited. Also, the influence of age and preoperative performance level, are incompletely understood. The aim of this study was to investigate whether APR translates into sustained pain relief over a long-term follow-up in athletes undergoing APR. Furthermore, we analyzed if age influences clinical and functional outcome measures in APR.

**Methods:**

Between 1998 and 2010, 30 competitive and recreational athletes were treated with APR due to chronic refractory patellar tendinopathy. All data were analyzed retrospectively. Demographic data, such as age or level of performance prior to injury were extracted. Clinical as well as functional outcome measures (Swedish Victorian Institute of sport assessment for patella (VISA-P), the modified Blazina score, pain level following exercise, return to sports, and subjective knee function were assessed pre- and postoperatively.

**Results:**

In total, 30 athletes were included in this study. At follow-up (8.8 ± 2.82 years), clinical and functional outcome measures such as the mean Blazina score, VISA-P, VAS, and subjective knee function revealed significant improvement compared to before surgery (*P* < 0.001). The mean time required for return to sports was 4.03 ± 3.18 months. After stratification by age, patients younger than 30 years of age yielded superior outcome in the mean Blazina score and pain level when compared to patients ≥30 years (*P* = 0.0448). At 8 years of follow-up, patients yielded equivalent clinical and functional outcome scores compared to our previous investigation after four years following APR.

**Conclusion:**

In summary, APR can be regarded a successful, minimally invasive, and sustained surgical technique for the treatment of patella tendinopathy in athletes. Younger age at surgery may be associated with improved clinical and functional outcome following APR.

**Electronic supplementary material:**

The online version of this article (10.1186/s12891-017-1851-3) contains supplementary material, which is available to authorized users.

## Background

Patellar tendinopathy (PT) is a common pathology in athletes performing repetitive jumping sports such as basketball and volleyball [1–4]. Recent studies suggest PT to emerge as an overuse of the knee joint extension mechanism and prolonged repetitive mechanical stress [4–15]. Athletes typically complain about anterior knee pain, leading to a reduction of exercise load and decline in the level of competition [1]. Temporary suspension of exercise combined with physiotherapy sufficiently reliefs pain in the majority of cases [4, 16–20]. However, if athletes continue to exercise, as often observed in professional athletes, consecutive inflammation of the patellar tendon can lead to degenerative alterations affecting the inferior patellar pole [21, 22]. Once patients remain symptomatic, conservative treatment approaches may fail resulting in 10% of athletes choosing to undergo surgery [6, 18]. Both, open and arthroscopic surgical modalities proved to be effective for the treatment of chronic-refractory PT and the majority of patients are able to return to sports [20]. However, it is unclear if preoperative performance levels can be achieved. Also, long-term data analyzing outcome of this patient cohort is still minimal. Ferretti and coworkers investigated patient outcome in competitive athletes following open surgery with a minimum follow-up of 5 years and demonstrated excellent results in 70% of the patients [16]. Pascarella et al. performed an arthroscopic debridement of the Hoffa’s body posterior to the patellar tendon and excision of the lower patellar pole and showed a failure rate after 3 years follow-up of 9.6% but still good and excellent results after a follow up of 5 and 10 years [19]. Maffulli et al. performed an open resection of tendinopathic tissue and analyzed clinical outcome at 7 years follow-up yielding in excellent results in more than 80% of cases [18]. Nevertheless, arthroscopic techniques are currently favored by the majorities of surgeons due to less approach-related morbidity and faster rehabilitation [21–23].

In 2006, Ogon and coworkers presented the technique of an arthroscopic patellar release (APR) [24]. To assess APR’s mid- and long-term results, Maier et al. recently followed patients for a mean of 4.4 years [25]. The majority of athletes (76.7%) were able to perform at previous sports levels.

Return-to-sports rates present a highly relevant parameter that is closely associated with success or failure after surgery.

In order to assess if APR remains successful for the treatment of chronic patellar tendinopathy in athletes in the long-term, we followed up on this cohort of patients. *Additionally, we investigated whether age at surgery influences clinical and functional outcome following APR within a sub-analysis of our study population.*

## Methods

### Study population

**Fig. 1:**
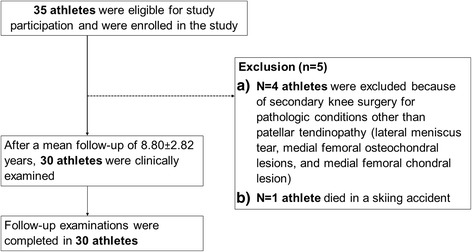
Study flow chart

We conducted a retrospective, single-center cohort study evaluating the clinical and functional outcomes of 30 athletes undergoing APR due to chronic-refractory, patellar tendinopathy between the years of 1998 and 2010. Inclusion criteria were age > 18 years, active sport participation, a minimal period of 6 months of conservative treatment, and postoperative follow-up period >5.6 years (Fig. 1). All patients were referred to our department for surgical treatment due to failed conservative therapy. Non-surgical treatment included eccentric physiotherapy for at least 6 months, oral non-steroidal anti-inflammatory drugs, extracorporeal shockwave therapy, and a maximum of 3 ultrasound-guided peritendinous corticosteroid injections.

### Preoperative diagnostics

All patients were examined by the same orthopedic surgeon (senior author) who had extensive experience in the treatment of PT. Patients presenting abnormal signal intensity on MRI, implying partial rupture of the proximal patellar tendon and concomitant intra- or extra-articular knee joint pathologies (i.e. patellofemoral malalignment/maltracking, chondral lesions >1° according to the International Cartilage Repair Society, meniscal tears and ligamentous injuries) were excluded from this study. Further exclusion criteria were detraction of sports performance and/or sports cessation for reasons other than patellar tendinopathy (e.g. secondary injuries of the affected extremity), incompliance, and/or use of performance enhancing drugs. Preoperatively, patient history was documented and a standardized clinical examination was performed. Radiographic assessment included conventional radiographs (anteroposterior / lateral knee views and axial patellofemoral views), ultrasonography, and magnetic resonance imaging (MRI) of the symptomatic knee joint to exclude intra- and extraarticular co-pathologies. Preoperative MRI evaluation of the patellar tendon and surrounding tissues were conducted utilizing T1, T2, and proton density-weighted sequences as described previously [25–27]. Only if a preoperative peritendinous injection of either local anesthetics or corticosteroids had caused an entire short-term relief of pain, arthroscopic treatment was recommended.

### Surgical procedure

All surgeries were performed by the same orthopedic surgeon (senior author). The surgical procedure and postoperative rehabilitation program were performed as previously described [21, 25]. Briefly, the skin of the inferior patellar pole was marked according to the preoperatively assessed symptomatic anatomic region followed by installation of the anterolateral portal. A standardized diagnostic arthroscopy of all knee compartments excluded potential intraarticular co-pathologies. Typically, synovial hypertrophy surrounding the inferior patellar pole was frequently detected. Then, an arthroscopic ablation probe (AAP; OPES; Arthrex, Naples, FL, USA) was inserted for focal synovectomy throughout the inferior patellar pole and the surrounding proximal part of the tendon. Finally, hypertrophic elements of the infrapatellar fat pad (Hoffa) were resected thoroughly to prevent impingement of the inferior patellar pole during extension. Finally, AAP was utilized to denervate the transitional bony zone at the inferior patellar pole at the respective symptomatic regions. Neither resection of the patellar tendon nor bony decompression had been performed in this study.

### Assessment of clinical and functional outcome

Systematic follow-up examinations were performed by a senior resident of the Department of Orthopedic Surgery. Follow-up assessment included palpation of the inferior patella pole, one legged stance, and light squats. Additionally, the Victorian Institute of Sport Assessment questionnaire for patients with patellar tendinopathy (VISA-P; 0-100 points) and the modified Blazina score served as functional outcome scores [16, 20, 25]. The modified Blazina score functions as a pathology-specific outcome measure including five stages according to symptoms occurring at different levels of sports/activity (0 = no pain, 1 = pain after intense sports activity, 2 = pain at beginning of and after sports activity, 3 = pain during activity at a satisfactory level, 4 = pain during sports activity at a non-satisfactory level, and 5 = pain during daily activity. Moreover, athletes evaluated the affected knee’s subjective function via SANE score [25, 28]. The number of months until athletes were able to perform specific exercises without any or minimal pain was extracted [29]. At follow-up, patients were asked to evaluate their symptoms and performance during sports compared to before onset of symptoms. A scale ranging from 0% to 100% was used to subjectively assess function of the affected knee with reference to patient’s unaffected contralateral knee joint. The unaffected knee joint was assessed at 100%. In order to assess pain levels during exercise we utilized the Visual Analog Scale (VAS) ranging from 0 to 100 points (no pain –maximum pain). Finally, all athletes were asked to rate their performance level in relation to their pre-injury conditions at follow-up.

### Postoperative rehabilitation

Patients were discharged at the day of surgery or at the first postoperative day. Starting on the first postoperative day, physiotherapy was performed according to patients’ complains without any restrictions regarding the range of motion and/or weight bearing. Once patients were symptom-free, open kinematic chain exercises were initialized and performed at least for 2 weeks postoperatively in order to maintain and strengthen quadriceps muscles. After two weeks, we instructed patients to start closed kinetic chain exercises and resumed sports-specific training but abstained excessive force during knee extension (i.e. jumping, running, bench press). After four weeks, patients underwent light running exercises and resumed training sessions followed by complete return to sports after 6 weeks in case the athletes were able to participate and complete their training sessions without experiencing pain. Previous research suggests that a sufficient time of absence from training following APR has beneficial impact on postoperative outcome.

### Statistical analysis

Statistical analysis was performed using SAS, v9.3 (SAS Institute, Cary, North Carolina, USA) and SPSS, v21 (IBM, Armonk, NY, USA). Descriptive results are given as mean values with standard deviations (±) and/or ranges. A non-parametric Wilcoxon test was used to compare subgroups of patients. Categorical parameters were analyzed with the exact Fisher test. Unpaired T-test and Chi-Square test were utilized to determine significant differences between parameters. Statistical significance was considered for *p*-values < 0.05.

In order to assess, whether age at surgery influences clinical and functional outcome following APR, a sub-analysis of our study population was performed dividing the cohort either in patients younger than 30 years or ≥30 years of age, respectively (Tables 2 and 4).

### Ethical considerations

The study was approved by the local Ethics Committee of the University of Freiburg (protocol number: 584/16) and informed consent was obtained from all participating patients before surgery.

## Results

### Baseline characteristics

**Fig. 2:**
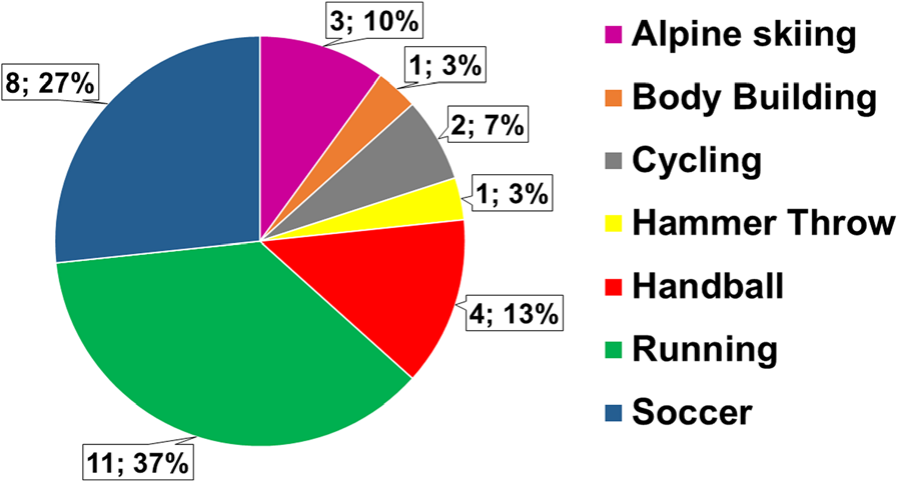
Study population stratified by type of sports

35 patients were eligible for study participation and were enrolled in the study (Fig. 1). Four patients were excluded because of secondary knee surgery for pathologic conditions other than patellar tendinopathy (lateral meniscus tear, medial femoral osteochondral lesions, and medial femoral chondral lesion). One patient died in a skiing accident. After a mean follow-up of 8.80 ± 2.82 years (range: 5.67 to 18 years), 30 athletes (26 male and 4 female) were clinically examined. In total, 11 patients performed on professional and 19 on amateur levels. Follow-up examinations were completed in 30 patients. As demonstrated in Fig. 2, sports involved were running (*n* = 11), soccer (*n* = 8), handball (*n* = 4), alpine skiing (*n* = 3), cycling (*n* = 2), hammer throw (*n* = 1), and body building (*n* = 1). None of the athletes experienced an injury of the index knee prior to surgery. Mean age of the study population at time of surgery was 28.23 ± 8.13 years (range: 16-49). No perioperative or surgical complications were experienced.

### Analysis of clinical and functional outcome

**Fig. 3:**
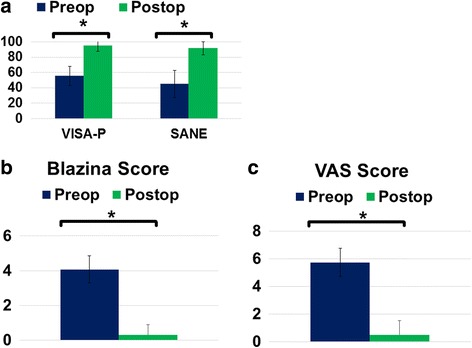
Clinical and Functional outcome of the study population. **a** Swedish Victorian Institute of sport assessment for patella (VISA-P) and Subjective Knee Function. **b** The modified Blazina score. **c** Visual Analog Scale (VAS) during exercise

All patients were clinically examined by the same senior orthopedic surgeon. Clinical evaluation at follow-up revealed significant improvements compared to before surgery (Table 1 and Fig. 3). From preoperatively to postoperatively, VISA-P score improved from 55.56±12.44 points to 95.43±8.06 points (*P* < 0.0001). At follow-up, 22 (73.33%) patients achieved excellent (91 to 100 points), 7 (23.33%) good (81 to 90 points) results. Only one (3.33%) patient presented with an unsatisfactory (70 points) outcome according to the VISA-P score. Additionally, functional assessment at follow-up via Blazina score revealed a significant improvement from 4.07±0.78 to 0.30 ± 0.60 compared to before surgery (*P* < 0.0001). At follow-up, 23 (76.67%) patients experienced no pain l (0 points), 5 (16.67%) felt minor pain after intense sports activity (1 point), and 2 (6.67%) patients had notable pain at the beginning and after exercising (2 points). Furthermore, we observed a significant improvement in the subjective knee function of our study population yielding 45.00 ± 17.76 preoperatively and 91.50 ± 8.72 at follow-up (*P* < 0.0001). As demonstrated in Table 2, fourteen patients (46.67%) evaluated their subjective knee function as excellent (91 to 100), 12 (40%) as good (81 to 90), 2 (6.67%) as satisfactory (71 to 80), and 2 (6.67%) as still unsatisfactory with a score of 70 in both cases. In the same way, pain levels declined from 5.73 ± 1.31 to 0.50 ± 1.01 at follow-up (*P* < 0.0001). A majority of 22 (73.33%) patients stated complete absence of pain.

**Table 1:** Clinical and functional outcome

Parameter	Preoperative	Postoperative	Significance
VISA-P score	55.56 ± 12.44	95.43 ± 8.06	*P* < 0.0001
Modified Blazina score	4.07 ± 0.78	0.30 ± 0.60	*P* < 0.0001
Pain (VAS)	5.73 ± 1.31	0.50 ± 1.01	*P* < 0.0001
Subjective Knee Function (SANE)	45.00 ± 17.76	91.50 ± 8.72	*P* < 0.0001
Mean Time Period for Return to Sports [months]	4.03 ± 3.18		

Mean ± SD; *P*-values ≤0.05 are considered statistically significant

**Table 2:** Clinical and functional outcome following arthroscopic patellar release stratified by age

Parameter	N	Percent	N	Percent	N	Percent
	All patients		<30y		≥30y	
VISA-P						
Excellent	1	3.33	1	5.56	0	0
Full	21	70.0	13	72.22	8	66.67
Good	7	23.33	4	22.22	3	25.00
Unsatisfactory	1	3.33	0	0	1	8.33
BlazinaBlazina						
0	23	76.67	16	88.89	7	58.33
1	5	16.67	1	11.11	3	25
2	2	6.67	0	0	2	16.67
VAS						
0	22	73.33	16	88.89	6	50
1	4	13.33	2	11.11	2	16.67
2	2	6.67	0	0	2	16.67
3	1	3.33	0	0	1	8.33
4	1	3.33	0	0	1	8.33
SANE						
Excellent	4	13.33	3	16.67	1	8.33
Full	10	33.33	7	38.89	3	25
Good	12	40	7	38.89	5	41.67
Satisfactory	2	6.67	1	5.56	1	8.33
Unsatisfactory	2	6.67	0	0	2	16.67

*Y* age in years, *VISA-P* Swedish Victorian Institute of sport assessment for patella, *Blazina* The modified Blazina score, *VAS* Visual Analogue Scale for knee pain, *SANE* Subjective knee function

Return to sports was achieved after 4.03 ± 3.18 months (range: 0.5-12 months) following APR. Overall, 24 athletes (80%) were able to return to their previous level of sports without any pain at follow-up. Four patients (13.33%) experienced minor restrictions during exercise. Two athletes (6.4%), a professional cyclist and a runner, felt notable pain at the onset and/or after sports activity. Postoperatively, all athletes were treated conservatively, since present complains were significantly improved when compared to before surgery. 80% of the athletes returned to their sports level within 6 months after surgery.

Recently, we have presented our outcome in patients with PT following APR due to failed conservative treatment at four years of follow-up (Table 3) [21]. Results of the present study demonstrate equivalent clinical and functional outcome after 8 years of follow-up compared to our previous assessment after 4 years, confirming the high success rate via APR (Table 3; *P* ≥ 0.05). We did not detect cases presenting a recurrence of symptoms or revision surgery.

**Table 3:** Comparison of clinical outcome between 4 and 8 years of follow-up following arthroscopic patellar release [21]

Parameter	4 years follow-up	8 years follow-up	Significance
VAS preoperatively	5.68 ± 1.08	5.73 ± 1.31	*P* = 0.862
VISA-P preoperatively	57.29 ± 11.35	55.07 ± 12.44	*P* = 0.997
Blazina preoperatively	4.03 ± 0.75	4.07 ± 0.79	*P* = 0.909
VAS postoperatively	0.57 ± 1.19	0.52 ± 1.02	*P* = 0.898
VISA-P postoperatively	95.07 ± 8.18	95.28 ± 8.15	*P* = 0.970
Blazina postoperatively	33 ± 0.66	0.31 ± 0.60	*P* = 0.854
Return to Sports (months)	4.35 ± 3.29	4.03 ± 3.23	*P* = 1.000
SANE	1.48 ± 0.85	1.37 ± 0.77	*P* = 0.880
Patient Satisfaction preoperatively	48.87 ± 18.15	45.33 ± 17.76	*P* = 0.996
Patient Satisfaction postoperatively	89.19 ± 12.05	90.00 ± 11.52	P = 0.997
Recurrence of symptoms	1.90 ± 0.30	1.87 ± 0.35	*P* = 0.654

*VISA-P* Swedish Victorian Institute of sport assessment for patella, *Blazina* The modified Blazina score, *VAS* Visual Analogue Scale for knee pain, *SANE* Subjective knee function

### Influence of age and level of performance

**Fig. 4:**
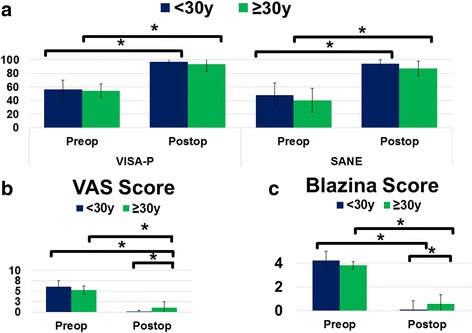
Clinical and Functional outcome of the study population stratified by age. **a** Swedish Victorian Institute of sport assessment for patella (VISA-P) and Subjective Knee Function. **b** Visual Analog Scale (VAS) during exercise. **c** The modified Blazina score

Stratifying our study population by age within a sub-analysis (Additional file 1), we found patients ≥30 years of age yielding inferior clinical and functional outcome assessed via Blazina (*P* = 0.0448) and VAS (*P* = 0.0122) score compared to patients younger than 30 years (Table 4 and Fig. 4). Postoperatively assessed SANE scores revealed differences between both age groups (<30 years: 94.17 ± 5.75 vs. ≥30: 87.50 ± 10.98), however these values did not reach statistical significance (*P* = 0.0819). Finally, patients achieving superior values in postoperative VISA-P and Blazina Score yielded better postoperative sports performance (*P* < 0.001) as well as patient satisfaction (*P* < 0.001) compared to patients with low VISA-P and Blazina Score.

**Table 4:** Clinical and functional outcome stratified by age

Parameter	< 30y	≥ 30y	Significance
VISA-P preoperative	56.33 ± 14.07	54.42 ± 10.0	*P* = 0.1297
VISA-P score postoperative	96.89 ± 5.75	93.25 ± 10.56	*P* = 0.6138
Modified Blazina score preoperative	4.22 ± 0.80	3.83 ± 0.72	*P* = 0.1760
Modified Blazina score postoperative	0.11 ± 0.32	0.58 ± 0.79	*P* = 0.0448
Pain (VAS) preoperative	6.06 ± 1.43	5.25 ± 0.97	*P* = 0.1259
Pain (VAS) postoperative	0.11 ± 0.32	1.08 ± 1.38	*P* = 0.0122
Subjective Knee Function (SANE) preoperative	48.06 ± 17.67	40.42 ± 17.64	*P* = 0.2665
Subjective Knee Function (SANE) postoperative	94.17 ± 5.75	87.50 ± 10.98	*P* = 0.0819
Mean Time Period for Return to Sports [months]	3.69 ± 2.60	4.54 ± 3.96	*P* = 0.9316

Mean ± SD; *P*-values ≤0.05 are considered statistically significant; *RTS* Return to Sports

Comparison FU (years) < 30: 8.51 ± 2.75; FU (years) ≥30: 9.24 ± 2.99; *p* = 0.2115

Age at surgery (years): <30: 23 ± 4; Age at surgery (years): ≥30: 36.08 ± 6.11

### Influence of MRI abnormalities

The present study is preceded by a similar study by our group that recently evaluated the incidence and influence of MRI abnormalities in patients suffering from PT [25]. Therefore, we have analyzed clinical and functional outcome measures and extracted preoperatively performed MRI data of the same study population (Table 5). Preoperatively, infrapatellar fat pad edema was observed more frequently among professional athletes compared to amateurs and recreational athletes (*P* < 0.001).

**Table 5:** Correlation between the preoperative level of sports and MRI abnormalities modified from Ogon et al. [1]

Parameter			MRI IFP edema		Total
			No	Yes	
Level of Sports	Professional	N (%)	2 (15.4%)	9 (50%)	11 (36.4%)
	Amateur	N (%)	10 (84.6%)	9 (50%)	19 (63.3%)
Total		N (%)	12 (100%)	18 (100%)	30 (100%)

*N* absolute number of patients, *%* relative number of patients; Data of *n* = 1 patient is missing

Significant correlations have been detected between the preoperative level of sports and the incidence of MRI pathologies (IFP edema). Higher incidences of MRI abnormalities among professionals and a homogenous distribution of MRI findings among amateurs have been observed (Chi-square-test: P < 0.001). *IFP* Infrapatellar fat pad

## Discussion

The present study sought to analyze long-term outcome of athletes undergoing arthroscopic patellar release due to chronic PT and evaluate factors influencing clinical and functional outcome. This study adds evidence that APR is a successful and sustained surgical therapy allowing for minimally invasive treatment of PT in patients with high functional demands. Younger age at surgery may be associated with improved clinical and functional outcome following APR.

### What is the optimal treatment for patellar tendinopathy in athletes?

Over the last years, different treatment methods for PT have been described. However, there is still no consensus towards the optimal strategy providing rapid and sustained relief of symptoms. Currently, strong evidence exists to recommend eccentric-squad-based training as conservative low-cost and low-risk treatment modality [30, 31]. Alternatively, platelet-rich plasma and shockwave therapy can also be performed as initial therapies [32]. Furthermore, heavy slow resistant training seems to be more successful compared to corticosteroid injections and pulsed ultrasound [33–35]. Approximately, 10% of patients are unresponsive to conservative treatments and thus undergo surgery mostly due to chronicity of symptoms [36]. In general, surgical therapy in PT is indicated for patients unresponsive to a minimum of 6 months of conservative treatment. Thus, evidence towards specific surgical techniques is still scarce [34]. Especially professional athletes are frequently hindered in their athletic ability and/or are even forced to quit their professional career because of the continuous experience of pain [3]. Prolonged duration of symptoms usually results in poorer outcome regardless of treatment modality [37]. Our group and others have recently demonstrated to achieve equivalent success rates (higher than 80%) via minimally invasive arthroscopic treatment in athletes with chronic PT [25, 38, 39]. Everhart and Muccioli found equivalent success rates between open and arthroscopic management of PT [26, 37]. However, the mean follow-up length of included studies was 15.2 ± 13.6 months. Therefore, long-term data on athletes with PT are highly valuable. Based on the results of the present study, we strongly recommend to perform minimally invasive techniques in athletes with PT if surgical therapy is considered, since the approach related morbidity can be reduced significantly compared to open approaches resulting in significantly faster recovery and return to sports [38]. Although open techniques yield equivalent VISA-P scores compared to arthroscopy, due to the very short length of follow-up, side effects, complications, delayed failure may be underrepresented. Compared to open surgical techniques APR has minimal risk for complications and demonstrates reduced postoperative pain and knee stiffness (never observed in our practice) [40]. Outcome of our study is comparable to previous studies on arthroscopic techniques with the major difference, that we have solely performed a patellar release without any tendon debridement and/or or osseous resection [39]. In general, arthroscopic techniques differ significantly in terms of the extent of patella tendon or bone resection [22, 23, 39, 41]. Here, we propose to solely resect neovascularizations and denervate the patella’s inferior pole. 80% of athletes were able to return to their previous sports performance without any pain at 8 years follow-up. The application of the success rate introduced by Coleman et al. (percentage of patients having excellent or good results) reveals an overall success rate in our study population after 8 years of 86.67% [42]. These values are similar to previously published outcome, ranging from 60% to 87.5% [16, 22, 42, 43]. Although presenting data of a fairly small study population, this investigation confirms our initial theory and previous studies that even through minimal invasive therapy (no bony and/or ligamentous debridement) significant and sustained (≥ 8 years follow-up) improvements are feasible with minimal risk. Therefore, we do not recommend any tendon or bone resection as this does not seem to translate into additional clinical or functional improvement.

### Predictors for success in arthroscopic patellar release

In general, surgery should be prevented in PT patients whenever possible, since current data propose satisfying clinical and functional outcome utilizing conservative therapies [37, 44]. Nevertheless, high recurrence rates have been observed especially in professional athletes (12%-27%), reflecting a high risk for performance loss, interruption/absence of exercise, and ultimately a premature end of the career [45]. To date, arthroscopic techniques have gained popularity due to their minimally invasive character while yielding equivalent success rates compared to open surgery. Arthroscopy can include shaving, release of posterior paratendon and bone denervation, and resection of the lower pole of the patella [22, 24, 46]. A frequently discussed question in this regard is, when and/or can success or failure be estimated? Our study suggests, that younger age (<30 years) may be associated with superior clinical (*P* = 0.0122) and functional outcome (*P* = 0.0448) compared to older patients (≥ 30 years) [25]. Patients enrolled into our study represent non-responders of conservative treatment presenting severe and chronic symptoms. Rio and co-workers recently demonstrated that jumping athletes with PT experience elevated corticospinal excitability for the rectus femoris muscle compared with healthy controls and people with other anterior knee pain, indicating specific pain behavioral neurological alterations in PT patients [47]. We assumed, that a longer duration of symptoms would (especially in our study population) lead to inferior outcome measures as recently published. Nevertheless, the vast majority of our patients yielded good clinical and functional outcome despite the long history of chronic pain. Risk factors for the development and/or recurrence of PT are training amount, intensity, age, BMI, weight, waist-to-hip ratio, leg length difference, hamstring flexibility, quadriceps strength, and vertical jump performance, and high total exposure [48]. Therefore, besides conducting the above-mentioned therapies, reducing body weight, increasing upper leg-flexibility and quadriceps strength and the use of orthotics may be beneficial in PT [45, 49]. However, the general evidence of these risk factors remains low. Based on our data, we may propose, that satisfying clinical and functional outcome after 4 years following APR can be maintained without having a singly revision case in our study population. This finding has highly significant implications for daily practice since APR is a true minimal invasive soft tissue procedure without any bony resection and the associated complications. Consequently, we can conclude that minimal invasive APR allows for sufficient and sustained long-term cure in athletes with failed conservative treatment of chronic patella tendinopathy. Furthermore, APR leads to equivalent clinical and functional outcome compared to other conservative or surgical treatment modalities.

### Limitations and strengths

Our study is associated with several limitations. Firstly, a lack of a control group, intermediate postoperative and/or follow-up examinations (to assess clinical and functional outcome at earlier postoperative time points) must be mentioned. Also, with a total of 30 patients our study population is small. Furthermore, extensive demographic data was lacking and did not allow for further sub-analysis. Our study population was biased due to the selection criteria. Patients, included in the study consisted of non-responders to conservative therapy. Interestingly, even in this patient cohort (in which initial treatment has failed) significant improvements on all clinical and functional scores could be achieved. Our analysis provides long-term outcome with a minimum follow-up of 8 years, which is highly valuable for surgeons and physiotherapists. Additionally, 37% of our patients were professional athletes indicating excellent outcome in patients with highest functional demands. As many studies on surgical treatment of PT lack significant methodological quality, we have incorporated the guidelines proposed by Coleman and co-workers, including the study design (prospective study), predefined patient inclusion / exclusion criteria, standardized algorithms for surgery and rehabilitation, and utilization of well-established clinical- and functional outcome measures [42]. To our knowledge, there are no previous reports on the clinical and functional outcome of APR in PT patients after such a long follow-up.

## Conclusion

APR allows for sufficient and sustained pain relief in athletes with chronic PT and can be utilized as minimally invasive low-risk surgical technique when conservative treatment fails. Younger age at surgery may be associated with improved clinical and functional outcome following APR in athletes.

## Additional file

Additional file 1: Table S1. Clinical and functional outcome following arthroscopic patella release stratified by age. **Table S2.** Correlation between the preoperative level of sports and previous surgeries. **Table S3.** Correlation between the preoperative level of sports and MRI abnormalities. **Table S4.** Comparison of clinical outcome between 4 and 8 years of follow-up following arthroscopic patella release. (DOCX 22 kb)

## Abbreviations

APR: Arthroscopic patellar release
BME: Bone marrow edema
IFP: Infrapatellar fat pad
MRI: Magnetic resonance imaging
PT: Patellar tendinopathy
SANE: Singular assessment numeric evaluation
VAS: Visual analog scale
VISA-P: Swedish Victorian Institute of sport assessment for patella
