# Supplementary material for: Arthroscopic patellar release for treatment of chronic symptomatic patellar tendinopathy: long-term outcome and influential factors in an athletic population
Source: BMC Musculoskelet Disord. 2017 Nov 22;18:486. doi: 10.1186/s12891-017-1851-3 (PMC5700547; doi:10.1186/s12891-017-1851-3)
Supplement: Additional file 1: Table S1. — Clinical and functional outcome following arthroscopic patella release stratified by age. Table S2. Correlation between the preoperative level of sports and previous surgeries. Table S3. Correlation between the preoperative level of sports and MRI abnormalities. Table S4. Comparison of clinical outcome between 4 and 8 years of follow-up following arthroscopic patella release. (DOCX 22 kb) [file 12891_2017_1851_MOESM1_ESM.docx]

**Supplementary**

**Supplementary Table 1. Clinical and functional outcome following arthroscopic patella release stratified by age**

| **Parameter** | **N** | **%** | **N** | **%** | **N** | **%** |
| --- | --- | --- | --- | --- | --- | --- |
| **VISA-P** | **All patients** | | **<30y** | | **≥30y** | |
| Excellent | 1 | 3.33 | 1 | 5.56 | 0 | 0 |
| Full | 21 | 70.0 | 13 | 72.22 | 8 | 66.67 |
| Good | 7 | 23.33 | 4 | 22.22 | 3 | 25.00 |
| Unsatisfactory | 1 | 3.33 | 0 | 0 | 1 | 8.33 |
|  |  |  |  |  |  |  |
| **Blazina** | **N** | **%** | **N** | **%** | **N** | **%** |
|  | **All patients** | | **<30y** | | **≥30y** | |
| 0 | 23 | 76.67 | 16 | 88.89 | 7 | 58.33 |
| 1 | 5 | 16.67 | 1 | 11.11 | 3 | 25 |
| 2 | 2 | 6.67 | 0 | 0 | 2 | 16.67 |
|  |  |  |  |  |  |  |
| **VAS** | **N** | **%** | **N** | **%** | **N** | **%** |
|  | **All patients** | | **<30y** | | **≥30y** | |
| 0 | 22 | 73.33 | 16 | 88.89 | 6 | 50 |
| 1 | 4 | 13.33 | 2 | 11.11 | 2 | 16.67 |
| 2 | 2 | 6.67 | 0 | 0 | 2 | 16.67 |
| 3 | 1 | 3.33 | 0 | 0 | 1 | 8.33 |
| 4 | 1 | 3.33 | 0 | 0 | 1 | 8.33 |
|  |  |  |  |  |  |  |
| **SANE** | **N** | **%** | **N** | **%** | **N** | **%** |
|  | **All patients** | | **<30y** | | **≥30y** | |
| Excellent | 4 | 13.33 | 3 | 16.67 | 1 | 8.33 |
| Full | 10 | 33.33 | 7 | 38.89 | 3 | 25 |
| Good | 12 | 40 | 7 | 38.89 | 5 | 41.67 |
| Satisfactory | 2 | 6.67 | 1 | 5.56 | 1 | 8.33 |
| Unsatisfactory | 2 | 6.67 | 0 | 0 | 2 | 16.67 |

**Y:** age in years; **VISA-P**: Swedish Victorian Institute of sport assessment for patella; **Blazina**: The modified Blazina score; **VAS**: Visual Analogue Scale for knee pain; **SANE**: Subjective knee function

**Supplementary Table 2. Correlation between the preoperative level of sports and previous surgeries**

| **Parameter** | | | **Previous Surgery** | | **Total** |
| --- | --- | --- | --- | --- | --- |
|  |  |  | **No** | **Yes** |  |
| Level of Sports | Professional | N (%) | 6 (24%) | 4 (66.7%) | 10 (31.3%) |
|  | Amateur | N (%) | 13 (52%) | 2(33.3%) | 15 (46.9%) |
|  | Hobby | N (%) | 6 (24%) | 0 (0%) | 6 (18.8%) |
| Total | | N (%) | 25 (100%) | 6 (100% | 32 (100%) |

**N:** absolute number of patients; **%:** relative number of patients; Data of n=1 patient is missing

The level of sports performed preoperatively (i.e. professional football player vs. hobby) correlates significantly with the history of previous surgeries (Chi-square-test: P<0.001).

**Supplementary Table 3. Correlation between the preoperative level of sports and MRI abnormalities modified from Ogon et al. [1]**

| **Parameter** | | | **MRI Patella edema** | | **Total** |
| --- | --- | --- | --- | --- | --- |
|  |  |  | **No** | **Yes** |  |
| Level of Sports | Professional | N (%) | 2 (15.4%) | 8 (44.4%) | 10 (31.3%) |
|  | Amateur | N (%) | 8 (61.5%) | 7 (38.9%) | 15 (46.9%) |
|  | Hobby | N (%) | 3 (23.1%) | 3 (16.7%) | 6 (18.8%) |
| Total | | N (%) | 13 (100%) | 18 (100% | 32 (100%) |

**N:** absolute number of patients; **%:** relative number of patients; Data of n=1 patient is missing

Significant correlations have been detected between the preoperative level of sports and the incidence of MRI pathologies (patella edema). Higher incidences of MRI abnormalities among professionals and a homogenous distribution of MRI findings among amateurs have been observed (Chi-square-test: P<0.001).

**Supplementary Table 4. Comparison of clinical outcome between 4 and 8 years of follow-up following arthroscopic patella release [21]**

| **Parameter** | **4 years follow-up** | **8 years follow-up** | **Significance** |
| --- | --- | --- | --- |
| VAS preoperatively | 5.68±1.08 | 5.73±1.31 | P=0.862 |
| VISA-P preoperatively | 57.29±11.35 | 55.07±12.44 | P=0.997 |
| Blazina preoperatively | 4.03±0.75 | 4.07±0.79 | P=0.909 |
| VAS postoperatively | 0.57±1.19 | 0.52±1.02 | P=0.898 |
| VISA-P postoperatively | 95.07±8.18 | 95.28±8.15 | P=0.970 |
| Blazina postoperatively | 33±0.66 | .31±0.60 | P=0.854 |
| Return to Sports (months) | 4.35±3.29 | 4.03±3.23 | P=1.000 |
| SANE | 1.48±0.85 | 1.37±0.77 | P=0.880 |
| Patient Satisfaction preoperatively | 48.87±18.15 | 45.33±17.76 | P=0.996 |
| Patient Satisfaction postoperatively | 89.19±12.05 | 90.00±11.52 | P=0.997 |
| Recurrence of symptoms | 1.90±0.30 | 1.87±0.35 | P=0.654 |

**VISA-P**: Swedish Victorian Institute of sport assessment for patella; **Blazina**: The modified Blazina score; **VAS**: Visual Analogue Scale for knee pain; **SANE**: Subjective knee function
